# Enhancing skin-implant integration in lower-limb transcutaneous prostheses: From interface biology to bioactive, antimicrobial and cell-based strategies

**DOI:** 10.1016/j.jot.2026.101145

**Published:** 2026-06-12

**Authors:** Ilaria Sergio, Rosalaura Caforio, Laura Sercia, Giuseppe Gigli, Francesca Gervaso, Alessandro Polini, Francesca Scalera

**Affiliations:** a CNR NANOTEC—Institute of Nanotechnology, C/o Campus Ecotekne, Via Monteroni, Lecce, 73100, Italy; b Tecnomed Puglia – Technopole for Precision Medicine (Biotech Lecce Hub), C/o Campus Ecotekne, Via Monteroni, Lecce, 73100, Italy; c Dipartimento di Ingegneria Dell'Innovazione, Università Del Salento, Campus Ecotekne, Via Monteroni, Lecce, 73100, Italy; d Dipartimento di Medicina Sperimentale, Università Del Salento, Campus Ecotekne, Via Monteroni, Lecce, 73100, Italy

**Keywords:** Amputation, Skin, Prostheses and implants, Infections, Peptides, Fibroblasts

## Abstract

Transcutaneous prosthetic systems, which directly connect an external limb prosthesis to the skeleton, offer substantial biomechanical and clinical advantages over conventional socket-based devices. By eliminating the soft tissue loading inherent to the socket interfaces, these systems enable improved load transfer, comfort, and more natural sensory feedback through direct skeletal attachment. However, their widespread clinical adoption remains limited by complications at the skin–implant interface, including infection, marsupialization, and epithelial down-growth.

Advances in surface engineering, particularly nanoscale modifications, have demonstrated a critical capacity to both promote host cell adhesion and direct macrophage polarization, thereby addressing epithelial sealing and chronic inflammation. Bioactive coatings incorporating extracellular matrix proteins or adhesion peptides have been designed to replicate dermal–epidermal interactions and reinforce epithelial anchorage. In parallel, antimicrobial strategies employing antibiotics, peptides, or metal-based coatings have been developed to counteract bacterial colonization of the stoma. Moreover, cell-based therapies using fibroblasts and mesenchymal stem cells have shown promise in supporting dermal integration and modulating local inflammation. Despite these advances, durable and infection-resistant skin–implant integration remains unresolved, indicating that long-term clinical success will require integrated, multifunctional interfaces capable of simultaneously supporting soft-tissue sealing, infection control, immunomodulation and mechanical stability.

**The translational potential of this article:**

The development of transcutaneous prostheses designed to enhance skin–implant integration in lower-limb amputees holds substantial translational promises, with implications that extend from individual patient outcomes to broader healthcare systems and biomedical innovation pipelines. Although osseointegrated prosthetic systems have demonstrated improved mobility and enhanced comfort, compared with traditional socket-based devices, their widespread clinical adoption remains limited by complications at the percutaneous interface. Addressing these challenges through bio-hybrid interface creates a realistic pathway toward an infection-resistant and durable skin attachment.

From a translational perspective, this strategy is gaining increasing interests due to its practical feasibility: several of its core elements, including surface-modified titanium, antimicrobial coatings, and bioactive agents, are supported by existing regulatory pathways. This enables stepwise innovation within well-defined approval frameworks without the need to create entirely new regulatory categories.

Next-generation implants could build upon clinically accepted materials while incorporating advanced surface functionalities that promote epithelial sealing, dermal integration, and controlled immune modulation. Smart, bioresponsive surfaces capable of releasing antimicrobial or anti-inflammatory agents in response to bacterial colonization further strengthen the clinical value proposition by directly addressing infection risk, one of the primary causes of implant failure and revision surgery. Reducing these complications, improving the biological seal at the skin–implant junction, is not merely a solution to a complication: it is an initial enabling technology for the next generation of smart prostheses, that would integrate sensors and neural communication systems, and decrease long-term healthcare costs associated with hospitalizations, antibiotic therapy, and surgical revisions, thereby reducing the overall cost of healthcare system.

Successful clinical translation will depend on multidisciplinary collaboration, robust preclinical modelling of the skin interface, and carefully designed clinical trials that evaluate both biological integration and functional outcomes. Enhanced skin–implant integration could shift transcutaneous prostheses from a niche intervention to a broadly adopted standard of care, redefining long-term rehabilitation strategies for individuals living with limb loss.

## Graphical abstract

**Figure:**
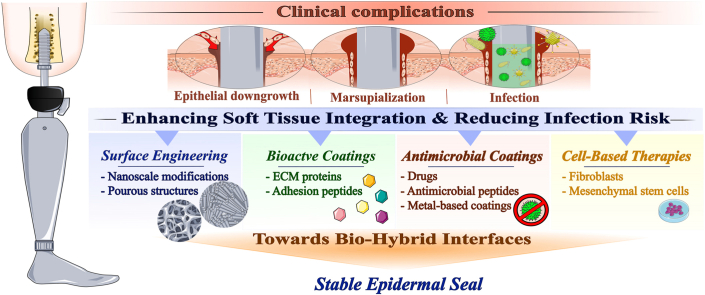

## 1 Introduction

Limb amputation severely impairs mobility and quality of life, and conventional socket-based prostheses often exacerbate these limitations due to poor load tolerance of residual soft tissues [[1], [2], [3]]. Recurrent skin irritation, ulceration and excessive perspiration are among the most common complications, affecting up to 70% of amputees in clinical surveys [4,5]. These issues frequently lead to reduced prosthesis use, impaired comfort, and diminished quality of life [3,6].

To overcome these limitations, direct skeletal attachment systems were introduced to bypass soft-tissue loading and improve load transfer between bone and prosthesis [3,7]. While early attempts at percutaneous prosthetic fixation demonstrated successful osseointegration, they also revealed a major obstacle: persistent failure of soft-tissue sealing, often leading to infection and device removal within months [[8], [9], [10], [11]]. The Brånemark OPRA system represented a turning point by adapting dental titanium implant principles to limb prosthetics through a two-stage surgical protocol ensuring controlled osseointegration [[12], [13], [14], [15]]. Long-term studies confirmed substantial results in mobility and body image, despite superficial infection rates near 70% and mechanical complications of ∼20% [[16], [17], [18]].

**Fig. 1:**
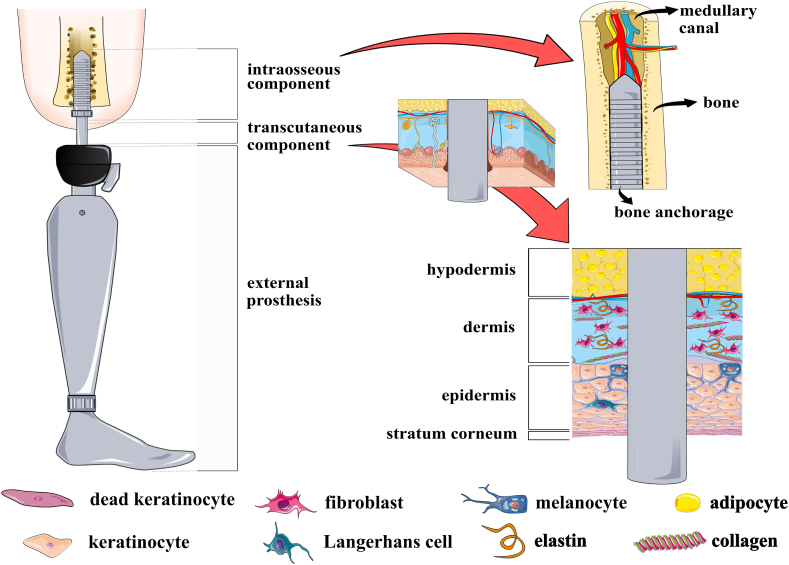
**Schematic representation of the Intraosseous Transcutaneous Amputation Prosthesis (ITAP) system**. The image shows the three functional system components and highlights the bone-implant and the skin-abutment interfaces. Adapted from Servier Medical Art (https://smart.servier.com/), licensed under CC BY 4.0 (https://creativecommons.org/licenses/by/4.0/).

To address the soft-tissue barrier, the Intraosseous Transcutaneous Amputation Prosthesis (ITAP) introduced a porous subdermal titanium flange, designed to promote dermal ingrowth and reinforce epithelial sealing—an evolution inspired by natural hard–soft junctions such as hooves and antlers [19]. The device consists of three main components: (i) a intramedullary stem (osseointegration region); (ii) a subdermal porous titanium flange (dermal anchorage region); and (iii) a transcutaneous abutment (epidermal interface) [3,20,21]. This architecture embodies two principles: direct bone-metal contact for efficient load transfer, and off-loading of residual skin from the compressive/shear stress typical of sockets [22,23] (Fig. 1).

Compared to conventional socket-based prostheses, ITAP implantation has been associated with improved body image [24], greater sitting comfort [25], improved hip mobility [26], easier fitting and removal [16], enhanced walking ability [24] and restored osseoperception, the ability to sense mechanical stimuli through the prosthesis [27].However, from a clinical standpoint, a successful ITAP application requires careful patient selection and surgical planning. Candidacy is typically restricted to amputees with severe socket intolerance or persistent dermatological complications at the stump, and clinical success largely depends on residual bone length and integrity of the surrounding soft-tissue envelope [17,28,29]. This aspect will be further explored in the following section.

Despite these significant functional gains and stringent clinical prerequisites, skin–implant interface remains the system's most critical failure-prone region [28,30]. Therefore, the skin–implant interface represents an important bio-mechanical challenge, as it is simultaneously exposed to severe shear stress and the convergence of bacterial proliferation and epithelial downgrowth. Clinical series report infection rates up to 30–40% in transcutaneous prostheses within 2–3 years, typically requiring antibiotic management [28,31].

**Fig. 2:**
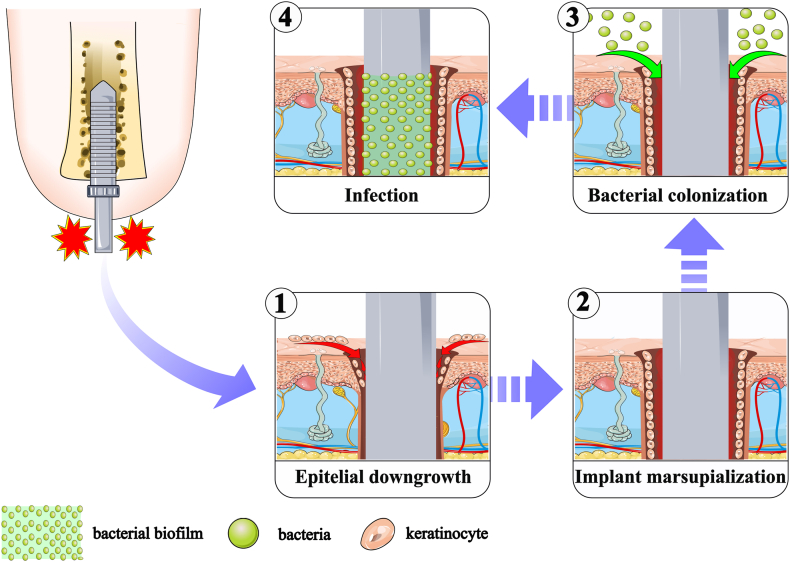
**Schematic representation of key failure modes at the skin–implant interface**. The image illustrates epithelial downgrowth—where inadequate early keratinocyte adhesion leads to migration of the epidermis along the abutment—and bacterial biofilm formation, driven by delayed epithelial sealing and surface colonization by *Staphylococcus aureus*. These events compromise the epidermal–dermal seal and initiate chronic inflammation, ultimately leading to infection and implant loosening. Image adapted from Servier Medical Art (https://smart.servier.com/), licensed under CC BY 4.0 ((https://creativecommons.org/licenses/by/4.0/).

This failure is fundamentally governed by the competitive process—termed the “race to the surface”—where early epithelial cell adhesion must outcompete bacterial colonization and subsequent biofilm formation to ensure long-term stability [19,[31], [32], [33], [34], [35]] (Fig. 2).

From a biological perspective, establishing a durable percutaneous seal requires the reconstitution of the epidermis–basement membrane–dermis triad, the body's natural defence against microbial invasion. This process is highly dependent on specific molecular anchoring: basal keratinocytes adhere to Laminin-332 rich basement membrane via α6β4 integrin and BP180-based hemidesmosomes. The structural integrity of this junction is further reinforced by the underlying collagen IV (basement membrane) and the collagen VII anchoring fibrils that tether the entire structure to the dermis [[36], [37], [38], [39], [40], [41], [42], [43]]. Although these cues have been successfully leveraged in dental implantology [33,[44], [45], [46]], direct translation of this approach to lower-limb stoma is limited by substantial anatomical and biomechanical differences, including greater soft-tissue thickness, higher shear forces during the gait, and distinct local microenvironments.

**Fig. 3:**
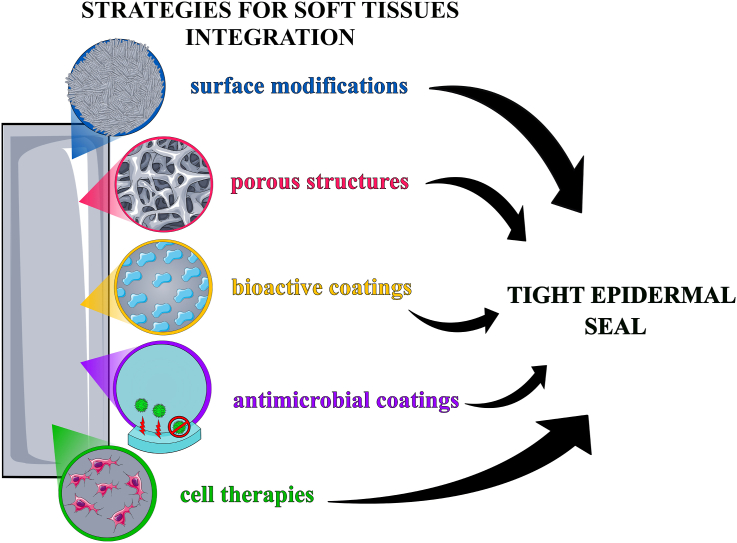
**Overview of complementary strategies designed to enhance skin–implant integration**. The techniques include surface and nano-topographical modifications, porous architecture, bioactive and antimicrobial coatings, and cell-based therapies. Image adapted from Servier Medical Art (https://smart.servier.com/), licensed under CC BY 4.0 ((https://creativecommons.org/licenses/by/4.0/).

The persistent vulnerability of the stoma has therefore driven the exploration of several bio-integrative strategies, which can be broadly categorized into five categories: surface nanoengineering, porous architectures, bioactive coatings, antimicrobial functionalization, and cell-based therapies. These approaches operate across distinct spatial and temporal scales, collectively aiming to promote stable tissue integration while conferring infection resistance (Fig. 3).

The following sections review these approaches, emphasizing emerging bio-hybrid strategies that integrate mechanical, biochemical and cellular cues to achieve long-term clinical stability.

## 2 Clinical limitations of current ITAP management

The successful clinical implementation of the ITAP system also relies on rigorous preoperative assessment confirming adequate residual bone length and soft tissue quality, as radiographic bone density frequently decreases following disuse of the residual limb. However, even with optimal patient selection and the use of standardized surgical protocols, current clinical management focuses primarily on macro-scale stability, often overlooking the microscopic biological sealing required at the skin–implant interface. Whether a single-stage or a two-stage surgical approach is adopted—separating bone healing from stoma creation—neither strategy inherently addresses the biological vulnerability of the transcutaneous exit site.

Postoperative care, which includes *Staphylococcus*-targeted prophylaxis and intensive rehabilitation over six months, remains largely reactive, managing the complications as they arise rather than preventing failure at its source.

Consequently, despite these rigorous clinical efforts, persistent infection rates of 30–40% remain high, underscoring the fundamental “pain points” of ITAP: the apical migration of junctional epithelium (downgrowth), the formation of infection-prone pockets, and chronic mechanical instability of the stoma under loading conditions. This clinical bottleneck demonstrates that long-term outcomes are constrained by incomplete soft-tissue integration [47] providing a clear rationale for the interface-engineering strategies discussed in the following sections, which aim to complement clinical protocols by directly improving epithelial anchorage and antimicrobial defense.

## 3 Engineering strategies for stable skin-implant integration

### 3.1 Surface micro-nanotopography for interface modulation

Modulation of the abutment surface topography represents one of the earliest strategies explored to enhance soft-tissue integration at the skin-implant interface. These approaches can act either at the cell-material interface level or within the bulk of material by operating across different length scales, consequently induce different biological effects [48]. In this context, surface topography allows to modulate cell adhesion, immune response, and bacterial interactions through nano- and micro-scale surface modification, without altering the bulk structure of the implant.

#### 3.1.1 Effects of nanotopography on cell adhesion

Surface roughness is a critical factor influencing the biocompatibility of metallic implants as micro- and nanoscale irregularities create a surface texture that directly modulate cell anchorage, spreading and proliferation. In this context, it is well established that surface morphological modifications can significantly enhance the biological response across various biocompatible materials. For instance, Bashir et al. [49] demonstrated that nanosecond-pulsed laser surface texturing on SS316L, Co-Cr and Ti6Al4V alloys effectively promotes cell adhesion and growth. Among these, stainless steel (SS316L) exhibited the most favourable roughness value (Ra = 0.897 μm), which was correlated with the highest cell density. Early comparative studies [42] demonstrated that, while smoother finishes (Ra <0.1 μm) promoted early keratinocyte spreading, the microscale roughness (Ra ≈0.3-2 μm) had a predominantly early, transient effect on cell behavior, largely diminishing after 72 h. This limitation shifted attention toward nanometer scale features, such as titania nanotube arrays [50], which supported significantly higher keratinocyte density than conventional titanium. Significantly, the cell densities achieved on nanostructured titanium alone were comparable to those achieved by titanium functionalized with covalently immobilized fibroblast growth factor-2 (FGF-2), suggesting that nanotopography may act as a growth-factor-sparing strategy [50]. Similarly, nanospikes and nanopores created by alkali-heat treatment [51] enhanced dermal fibroblast adhesion, cytoskeletal organization, and critically, robust extracellular matrix (ECM) production. By day 28, collagen fibers were observed to resist detachment treatments applied to mimic mechanical and inflammatory stress, highlighting the role of nanofeatures in providing mechanical stability [52]. Consistently, anodically generated nano-pits (50–60 nm), by modulating surface roughness, wettability, and protein adsorption, promote fibroblast recognition, attachment, and proliferation to stainless steel, proving optimal biological response [53].

#### 3.1.2 Immunomodulatory role of nanotopography

Beyond promoting cells adhesion, surface topography also plays a crucial role in modulating the immune response at the host-implant interface, a fundamental aspect for the long-term success of transcutaneous devices. It is increasingly recognized that nanoscale surface characteristics serve as physical cues that direct macrophage polarization, a concept that sits at the heart of the “race for the surface” between host cells and invading pathogens [20]. The capacity to direct macrophage polarization from the pro-inflammatory (M1) phenotype to the pro-regenerative and anti-inflammatory (M2) one has emerged as a key strategy to promote tissue integration and prevent the chronic foreign body reaction and fibrotic response that precedes epithelial downgrowth [54]. This is particularly critical since titanium itself can elicit proinflammatory and tissue-destructive responses in primary human, thereby reinforcing the need for nanotopographical cues capable of shifting macrophage behavior toward a pro-regenerative phenotype. Recent research has shown that nanotopographies (surface structures typically smaller than 100 nm) act as potent mechanotransduction signals, influencing macrophage polarization through cytoskeletal remodeling [55]. For example, Zhu and colleagues [56] observed that specific nanoscale honeycomb surfaces patterns of 90 nm significantly induce the anti-inflammatory M2 phenotype, creating a microenvironment favourable for integration. Similarly, the creation of super-hydrophilic TiO_2_ nanotube arrays on titanium has proven to significantly reduce the inflammatory M1 response (e.g., TNF- α) and increasing the reparative M2 polarization (e.g., IL-10) [57]. A systematic review of *in vitro* studies has also confirmed that titanium surfaces with micro/nano-scale roughness and hydrophilic surface chemistry are the most effective in inducing an anti-inflammatory macrophage phenotype [58]. Although the inflammatory response to metallic implants can be influenced by material composition, as demonstrated by differences among alloys such as Ti6Al4V, CoCrMo and stainless steel [59], these effects are often transient and associated with degradation processes. In contrast, increasing evidence suggests that surface nanotopography itself acts as a major determinant of macrophage behavior, independently of the underlying material. This concept is reinforced by studies on non-titanium-based materials, indicating that immunomodulation is not an exclusive feature of titanium chemistry. For instance, on nanopatterned polydimethylsiloxane (PDMS) substrates, Li et al. [55] showed that specific nanopillars and nanopits reduced M1-associated markers while increasing CD206 and other M2-associated genes, demonstrating that macrophage polarization can be directed by nanoscale geometry itself. Similarly, Wang et al. [60] reported that hydroxyapatite ceramics with nano-topography promoted the transition from M1 to M2 macrophages, reduced pro-inflammatory cytokine secretion, and attenuated tissue inflammation both *in vitro* and *in vivo*. In a soft-tissue-related model, Wu et al. [61] further showed that nanostructured zirconia surfaces modulated macrophage phenotype and, through conditioned media, enhanced gingival fibroblast proliferation and extracellular matrix production. Collectively, these findings support the view that surface topography itself is a key regulator of macrophage behavior, while material chemistry acts as a contributing, but not exclusive, determinant.

#### 3.1.3 Antibacterial and bactericidal effects of nanostructures

Beyond their effects on cell adhesion and immune modulation, nanotopographical surfaces also offer a promising strategy to reduce bacterial colonization, which is essential for the establishment of a stable biological seal [56].

*In vivo,* [50] hierarchical architectures combining nanoscale texture with an underlying microtopography significantly enhanced subcutaneous adhesion and reduced susceptibility to *Staphylococcus aureus* infection compared to smooth controls. Nanostructured surfaces have been shown to limit staphylococcal attachment, while simultaneously supporting favourable host-cell response [38,39,62].

Mechanistically, the antibacterial activity of these surfaces appears to depend, at least in part, on the mechanical interaction between bacterial cells and nanoscale surface features. In this regard, Sheu et al. [63], showed that different nanostructured patterns can induce mechanical stress on the bacterial cell wall, resulting in varying degrees of bacterial inhibition. These findings support the concept that nanoscale surface design can provide a physical, rather than purely chemical, route to antibacterial activity and thus represents a promising approach for improving implant resistance to bacterial colonization.

Taken together, these considerations suggest that the greatest translational potential lies in multiscale hierarchical architectures capable of combining favourable immune modulation, improved biological sealing, and antibacterial performance. Key representative studies are summarized in Table 1.

**Table 1:** Effects of surface topography and multiscale architectures on initial cell adhesion, immunomodulation and antibacterial activity.

Author (Year)	Material/Architecture	Model/System	Key Findings (Mechanisms and Implications)
Pendegrass et al. (2008) [42]	Ti Smooth vs. Micro-Roughness (Ra ≈0.3-2 μm)	*In vitro* (Keratinocytes)	Promotion of initial adhesion, emphasizing the need for nanoscale features in smoother surfaces (Ra <0.1 μm)
Zile et al. (2011) [50]	Titania Nanotube Arrays (Nanotubular Ti)	*In vitro* (Keratinocytes)	High keratinocyte density.
Yamada et al. (2016) [51]	Ti with Nano-Spikes and Nano-Pores (Alkali-heat treatment)	*In vitro* (Human dermal fibroblasts)	Enhanced dermal fibroblast adhesion and robust ECM production.
Gao et al. (2020) [57]	Super-hydrophilic TiO_2_ Nanotube Arrays (H2-TNTs)	*In vitro* (Macrophages)	Reduction of inflammatory M1 markers and rise of reparative M2 polarization to support osteogenesis.
Li et al. (2022) [55]	Nanopatterned Structures (Various)	*In vitro* (Macrophages)	Influence on macrophage phenotype by regulating cell shape and cytoskeletal remodeling.
Zhu et al. (2025) [56]	Hydrogenated TiO_2_ Nanoscale Honeycomb Surface Pattern (90 nm)	*In vitro* (Macrophages, *S. aureus*)	Induction anti-inflammatory M2 phenotype and provision NIR-responsive bactericidal properties.
Turner et al. (2025) [20]	Various Titanium Topography and Wettability	*In vitro* Co-culture (THP-1 Macrophages and *S. aureus*)	Modulation on balance between host immune cell adhesion and bacterial colonization.
Lindsay et al. (2020) [58]	Hierarchical Multiscale Architecture (Nano on Micro)	Rat (Subcutaneous implantation)	Enhanced subcutaneous adhesion and lower susceptibility to *S. aureus i*nfection compared to smooth controls.

Ultimately, while surface texturing is highly effective for bone-implant osseointegration, it appears insufficient for ensuring long-term soft-tissue stability. Although topographical cues effectively promote early cell adhesion, they cannot overcome the persistent mechanical mismatch between the rigid metallic component and the compliant surrounding skin. This bottleneck highlights that surface-level modifications alone are inadequate for the transcutaneous interface, necessitating the transition toward the three-dimensional structural strategies.

### 3.2 Role of porosity in soft tissue ingrowth

While surface topography enhances early cell adhesion, it does not fully ensure stable, long-term tissue integration particularly for the soft-tissue component. To overcome this limitation, researchers have investigated the effects of porous architectures on cell infiltration, ECM deposition and vascularization [[64], [65], [66], [67], [68], [69], [70]]. Hulbert et al. [71] demonstrated, in an *in vivo* canine model, that significant tissue infiltration occurred when pore diameters exceeded 100 μm. Building on this, Kuboki et al. [67] confirmed that only interconnected porous structures supported osteogenesis, providing the conceptual basis for exploiting similar principles for soft-tissue integration. Later, Pendegrass et al. [72] designed a porous flanged implants (700 μm pores) surgically inserted into the medial tibia of skeletally mature goats. Multiple surface configurations (machined, sandblasted, grooved, hydroxyapatite-coated, and diamond-like carbon) were compared between straight and flanged designs. After four weeks, histological analyses showed that hydroxyapatite-coated flanges promoted robust dermal tissue ingrowth and significantly reduced epithelial downgrowth relative to straight controls. In the same period, the concept was also explored in a pilot animal study by Pitkin et al. [65]. Using titanium pylons fabricated from sintered particles (30% porosity) they demonstrated deep fibrovascular infiltration into the porous structure, resulting in a more stable interface and significantly reduced inflammatory signs (i.e., abscess formation) compared with smooth titanium controls.

Driven by the necessity to define optimal structural parameters for long term stability, Chimutengwende et al. [66], developed laser-sintered porous titanium alloy flanges featuring 700 μm pore size, 300 μm strut size and 18% porosity. In a sheep model, these fully porous structures improved dermal attachment, supported vascularized tissue ingrowth, and reduced epithelial downgrowth compared with drilled ITAP flanges. Even when complete epithelial integration was not achieved, stable dermal anchorage was sufficient to prevent downgrowth, thereby expected to reduce bacterial colonization, confirming the role of porosity in favoring cell dominance in the “race to the surface.” The authors found that additional coatings (hydroxyapatite, silver, fibronectin) showed limited additional benefit compared to porosity alone. Follow-up studies further refined these findings, identifying that a 700 μm pore diameter and 300 μm strut thickness that provided the optimal critical balance between promoting vascularization and maintaining mechanical properties [1].

Extending this strategy to deep dermal anchorage, Jeyapalina et al. [73] demonstrated in a sheep model that porous titanium layers (pore size 300–400 μm, coating thickness 0.5 mm) achieved complete soft-tissue integration and effectively prevented both superficial and deep infections (0% vs. 25% in smooth Ti controls) over nine months. Histological analyses revealed dense, well-vascularized connective tissue penetrating the porous coating and forming a stable epidermal–dermal seal.

However, the porous architecture introduces a critical translational trade-off: while large, interconnected pores are essential for vascularized tissue ingrowth, they also serve as potential reservoirs for bacterial colonization and debris accumulation, making surface stability paramount. The long-term success under functional loading—common in lower-limb applications—relies heavily on managing this instability. Holt et al. [74] conducted a complementary long-term investigation under functional loading, implanting smooth and plasma-sprayed porous-coated titanium rods (Ra ≈2.8 μm vs. 0.6 μm) into the forelimbs of skeletally mature sheep for up to 12 months.

Quantitative analyses at 6, 9, and 12 months revealed that porous coatings significantly reduced epithelial downgrowth and increased the continuity of dermal attachment. Histological evaluation further showed progressive remodeling of peri-implant skin, with distinct morphological adaptations across the epidermal, subepidermal, and deep dermal layers. These changes were attributed to the gradual establishment of a mechanical stiffness gradient between the compliant soft tissue and the rigid implant surface, mitigating the compliance mismatch and reducing shear-induced regression, a key failure mechanism. Holt et al. also noted that immediate postoperative immobilization may enhance this adaptive process. These findings indicate that the clinical value of porous architectures depends on defining an “ideal porosity window” able to maximize vascularized tissue integration and robustness under load without increasing susceptibility to infection. Key representative studies are summarized in Table 2.

**Table 2:** Key studies on porous architectures: Optimization of geometry, deep tissue anchorage, and biomechanical trade-offs.

Author (Year)	Material/Architecture	Animal Model/System	Study Duration	Key Findings (Anchorage and Stability)
Kuboki et al. (1998) [67]	Porous HA Ceramics (Mechanistic Principles)	Rat (Subcutaneous implantation)	4 Weeks	Defined the structural requirement for bioactivity (porosity>100 μm and interconnection), establishing the conceptual basis for subsequent soft-tissue *ingrowth* studies.
Pitkin et al. (2006) [65]	Porous Ti Pylon (Sintered particles, 30% porosity)	Wistar Rat	4 Weeks	Demonstrated deep fibrovascular infiltration, which resulted in a stable interface and significant reduction of inflammatory signs (compared to smooth controls).
Chimutengwende et al. (2017) [66]	Porous Ti Flanges (700 μm pore, 300 μm strut)	Sheep (Tibia)	4 Weeks	Confirmed the dominant role of volume engineering in integration (*“race to the surface”*); the optimal geometry (700/300 μm) proved superior to functionalized coatings.
Jeyapalina et al. (2012) [73]	Porous Subdermal Barrier (300–400 μm)	Sheep (Forelimb amputation)	9 Months	Achieved complete soft-tissue integration via porous barrier, demonstrating effective long-term infection prevention (0% vs. 25% in smooth controls).
Holt et al. (2013) [74]	Porous-Coated Ti Rods under Functional Loading	Sheep (Forelimb amputation)	Up to 12 Months	Demonstrated that the porous coating establishes a mechanical stiffness gradient that minimizes shear-induced regression, a key failure mechanism under functional load.

### 3.3 Bioactive coatings to promote keratinocyte and fibroblast adhesion

While nanostructured and porous titanium surfaces enhance soft tissue integration, their intrinsically bioinert nature necessitates the introduction of biological cues to actively promote specific cellular adhesion and durable tissue anchorage [2]. To overcome this limitation, numerous studies have explored biofunctional coatings that mimic the natural dermal-epidermal interface. These strategies aim to replicate integrin-mediated interactions that regulate epithelial and dermal cell attachment by immobilizing ECM proteins such as fibrinogen [69], fibronectin [66,70,75] and laminin [51]. Alternatively, researchers utilize short peptide sequences derived from these proteins, such as RGD (derived from fibronectin/fibrinogen) and YIGSR motifs (derived from laminin) [68].

#### 3.3.1 E-cadherin functionalization: targeting epithelial specificity

The formation of the epithelial barrier is fundamental for long-term stability. Pendegrass et al. [5] pioneered the use of titanium surface functionalized with the extracellular domain (ECD) of E-cadherin, a key transmembrane component protein of adherens junctions that mediates keratinocyte–keratinocyte adhesion. The authors hypothesized that surface immobilized E-cadherin could mediate specific keratinocytes adhesion, thereby preventing epithelial downgrowth. Their results demonstrated a fourfold increase in β-catenin expression at the cell-substrate interface and a twofold to threefold increase in cell area compared with unmodified surfaces, confirming strong and specific cellular engagement.

In a subsequent study, Dehli et al. [76] functionalized titanium with E-cadherin-Fc fusion protein (E-cadherin fused with the Fc domain of human IgG) and Protein A (a surface protein found in the *S. aureus*'s cell wall, known for its IgG binding capacity) using polydopamine (PDA) as intermediate linker to enhance stability. This approach resulted in a robust coating, stable for up to two months in cell culture media. Functional assays confirmed that the immobilized E-cadherin-Fc protein significantly improved keratinocytes adhesion while simultaneously preventing adhesion and proliferation of the primary human dermal fibroblasts, which lack E-cadherin expression. Moreover, incorporation of Protein A into the coating significantly reduced keratinocyte migration in a wound-healing assay, underscoring its potential to act as a physical barrier against epithelial down-growth *in vivo*. However, despite the promising epithelial specific attachment, this study highlighted a critical limitation: the selective absence of fibroblast adhesion. This finding underscores the need for complementary or multifunctional surface modifications capable of supporting both dermal fibroblast and epithelial cell attachment, which is essential for comprehensive and stable soft tissue integration around percutaneous implant [76].

#### 3.3.2 ECM-derived protein coatings: promoting dermal integration

For effective dermal fibroblast attachment, ECM proteins have been widely investigated as bioactive coatings. Fibrinogen [69], fibronectin (Fn) [66,70,75], and laminin [51] are among the most studied, as they play central roles in mediating integrin-dependent cell adhesion and tissue anchorage, primarily through binding RGD motifs which promote cell adhesion and ECM deposition.

Wang et al. [77] covalently linked fibrinogen to PDA-coated titanium implants. After three weeks of subcutaneous implantation in rats, they observed reduced fibrous capsule thickness and enhanced neovascularization compared to uncoated controls, indicating improved histocompatibility and attenuated inflammatory response. *In vivo* test with titanium screws, further showed that fibrinogen-functionalized implants significantly reduced infection rates and epithelial downgrowth.

Fibronectin has also been explored, often in combination with hydroxyapatite (HA), to promote both soft tissue and potential bone anchorage. Pendegrass et al. [75] demonstrated that a HA-Fn composite coating led to a sevenfold increase in fibroblast adhesion after 24 h compared to HA control, suggesting a synergistic mechanism that effectively replicates the ECM-like microenvironment. Chimutengwende-Gordon et al. [66] corroborated these findings in a sheep model, finding that an HA-Fn composite coating showed the highest percentage of stable soft tissue attachment at the interface, thereby validating the translational potential of the synergistic approach.

More recently, Tao et al. [78] developed a multiscale bioactive coating that combines hydroxyapatite enriched with trace elements (Si, Sr, F) and ECM proteins (type I collagen and fibrinogen) covalently immobilized via a PDA interface on porous Ti-6Al-4V scaffolds. The PDA-mHA-Col I-Fg coating showed excellent stability and markedly improved both osteogenic and epithelial cell responses *in vitro*. Pre-osteoblasts and keratinocytes enhanced adhesion and proliferation, suggesting that these hybrid bioinorganic–bioprotein coatings can simultaneously promote osseointegration and soft tissue integration, a crucial step toward stable transcutaneous implant interfaces.

#### 3.3.3 Peptide-based strategies: high specificity and stability

Short peptide sequences derived from ECM proteins represent an innovative and highly specific approach. Unlike full-length proteins, peptides offer advantages in terms of enhanced stability, chemical purity, and cost-effective synthesis, while maintaining the specific integrin-binding motifs required for cell adhesion.

Ghribi et al. [68] demonstrated the potential of this strategy by covalently grafting the RGD (fibronectin) and YIGSR (laminin) peptides on titanium alloy substrate using a stable phosphonate linking arm. *In vitro*, RGD-functionalized surfaces enhanced dermal fibroblasts spreading and viability. Specifically, they observed that the YIGSR-functionalized surfaces significantly improved keratinocyte viability and spreading, demonstrating the ability of peptides to selectively enhance the adhesion and viability of competing cell types (fibroblasts and keratinocytes), which is required to reinforce the biological seal.

Beyond cell-specific adhesion, modular peptides provide a further versatile approach, as they are capable of both binding the implant surface and independently eliciting well-defined biological functions. Polini et al. [79] demonstrated that surface-binding peptide domains can ensure stable functionalization and durable anchoring to HA surfaces, while tuning the biological activity associated to cell adhesion, proliferation, and differentiation. Consistently, Yazici et al. [80] studied peptide-functionalized titanium substrates showing that short peptide sequences can modulate cell–material interactions in a sequence-dependent manner.

Collectively, studies on ECM-derived protein and peptide coatings highlight the potential of bioactive functionalization strategies to selectively enhance the initial adhesion and viability of keratinocytes and fibroblasts *in vitro*. Key representative studies are summarized in Table 3.

**Table 3:** Summary of bioactive coating strategies promoting keratinocyte and fibroblast adhesion on titanium-based transcutaneous implants.

Author (Year)	Bioactive Agent/Coating Type	Substrate & Linker	Cell/Model System	Key Finding & Critical Implication
Pendegrass et al. (2012) [5]	E-Cadherin ECD (Adherens Junction Protein)	Ti-6Al-4V	*In vitro* (Keratinocytes)	Achieved strong and specific keratinocyte adhesion (4x increase in β-catenin expression), validating E-cadherin as a target to prevent epithelial downgrowth.
Dehli et al. (2019) [75]	E-Cadherin-Fc Fusion Protein	Ti/Polydopamine (PDA)	*In vitro* (Keratinocytes, Fibroblasts)	Robust, stable coating. Improved Keratinocyte adhesion while selectively preventing fibroblast adhesion, highlighting a limitation: the need for multifunctional coatings.
Wang et al. (2021) [76]	Fibrinogen (ECM Protein)	Ti/Polydopamine (PDA)	Rat (Subcutaneous, Ti screws)	Reduced fibrous capsule thickness *in vivo* and improved integration. Fibrinogen-coated screws reduced infection and epithelial downgrowth.
Pendegrass et al. (2012) [74]	Fibronectin (Fn) + HA (ECM Protein Composite)	Ti/HA Coating	*In vitro* (Dermal Fibroblasts)	Sevenfold increase in fibroblast adhesion compared to HA alone, demonstrating the synergistic benefit of combining bioactive agents with osseointegrative coatings.
Chimutengwende-Gordon et al. (2017) [65]	Silanized Fn or HA-Fn Composite	Ti alloy (Flanges)	Sheep (Chronic Implantation)	HA-Fn composite showed the highest percentage of stable soft tissue attachment *in vivo*, corroborating the potential of composite ECM coatings for dermal anchorage.
Tao et al. (2025) [78]	HA-Col I-Fg Hybrid Coating (Type I Collagen + Fibrinogen)	Porous Ti-6Al-4V/PDA	*In vitro* (Osteoblasts, Keratinocytes)	Hybrid bioinorganic-bioprotein coating simultaneously enhanced osteogenic and epithelial responses, addressing the critical need for a dual osseointegration/soft tissue seal.
Ghribi et al. (2023) [67]	RGD & YIGSR Peptides (ECM-derived Motifs)	Ti alloy/Phosphonate Linker	*In vitro* (Fibroblasts, Keratinocytes)	Demonstrated the ability of peptide grafting to selectively enhance the adhesion and viability of competing cell types (fibroblasts by RGD, keratinocytes by YIGSR), reinforcing the biological seal.

Taken together, bioactive coatings offer the important advantage of introducing specific biological cues at the implant surface, thereby promoting selective epithelial and dermal cell responses that cannot be achieved by topographical design alone. Their main translational limitation, however, is that biological activity does not necessarily translate into durable interfacial performance, since coating retention may be compromised by repeated shear stress, gait-related loading, and the persistently moist environment of the transcutaneous interface. In this regard, Ao et al. [81] showed in a rabbit model that collagen/hyaluronic acid multilayers with improved coating stability coating stability achieved better tissue integration and closer implant contact than physically adsorbed counterparts, supporting the view that coating stability is a critical determinant of *in vivo* performance. At the same time, direct evidence on anti-delamination capacity and long-term mechanical endurance under clinically relevant dynamic loading remains limited. Future progress will therefore depend on the development of multifunctional and mechanically robust coating systems that combine biological specificity with stable anchoring under clinically relevant conditions.

## 4 Antimicrobial strategies for enhancing implant safety

Infections remain the most prevalent and clinically problematic factor compromising the long-term success of transcutaneous implants. To mitigate the risk, metals such as silver (Ag), copper (Cu) and zinc (Zn) have been extensively investigated for their inherent, broad spectrum antimicrobial properties. Their antibacterial effects rely on multiple simultaneous mechanisms, which may reduce the likelihood of resistance development. However resistance to these metals has been reported, and concerns potential cross-selection with antibiotic resistance genes [82]. These metals also differ substantially in their biological trade-off: silver exhibits the highest antibacterial efficacy, but also the narrowest therapeutic window and the highest toxicity even at relatively low concentration [83]; copper promotes bacterial killing through reactive oxygen species (ROS) generation with potential collateral damage to host tissues; zinc shows milder antibacterial activity, but a more favourable cytotoxicity profile [82]. A crucial translational challenge is therefore to balance antimicrobial efficacy, with host cells compatibility, particularly for keratinocytes and fibroblasts. The following sections examine how recent studies address this balance through optimized coating design, matrix engineering and controlled ion release.

### 4.1 Copper and zinc systems: ion- and ROS-mediated activity and biocompatibility constraint

The investigation into the use of Cu and Zn as dopants was systematically explored by Zhang et al. [[84], [85], [86]] through TiO_2_ coatings, aiming to enhance antibacterial activity while promoting fibroblast compatibility. Specifically, in 2016, they demonstrated that micro-arc oxidized (MAO) TiO_2_ coatings doped with Cu^2+^ ions improved fibroblast adhesion and proliferation *in vitro*, concurrently exhibiting dose-dependent antibacterial activity against *S. aureus*. Subsequently, in 2017, they expanded this approach to Zn-doped TiO_2_ coatings featuring an outer layer of H_2_Ti_5_O_11_·H_2_O (HTO) nanoarrays. While the initial antibacterial effects were primarily attributed to surface topography, subsequent investigations confirmed the contribution of Zn ion release to antimicrobial activity. Building on this work, Zhang et al. [86] developed co-doped TiO_2_ coatings containing both Cu and Zn, observing enhanced antibacterial effects through contact-killing and release-killing mechanisms. These findings, however, highlighted a crucial challenge: higher Zn concentrations promoted mitochondrial activity in mouse fibroblasts only up to a specific threshold, beyond which cytotoxicity emerged. These observations underscore the difficulty of balancing metal ion concentrations to achieve robust antimicrobial efficacy without compromising host tissue viability.

To mitigate this dose-dependent cytotoxicity of metal ions, Leng et al. [87] introduced a novel hydrogel-based approach using responsive release kinetics. They developed a composite coating comprising a ZnO nanoflower-like structure overlaid with a methacrylated gelatin (GelMA) and methacrylated hyaluronic acid (HAMA) hydrogel. The addition of HAMA allowed for the enzymatic degradation of the coating, specifically triggered by *S. aureus*-mediated hyaluronidase secretion. This controlled degradation exposed underlying ZnO regions, enabling targeted release of Zn ions and enhanced production of reactive oxygen species (ROS), both key mechanisms for antibacterial activity. Importantly, by reducing direct contact between ZnO and surrounding tissues, the hydrogel coating significantly improved fibroblast viability and promoted soft tissue ingrowth under physiological conditions. This strategy effectively leveraged a biological trigger to achieve on-demand, localized antibacterial action while greatly enhancing biosafety.

### 4.2 Silver systems: optimization of concentration and release profile

Parallel to the development of Cu and Zn system, the use of Ag coatings has been extensively explored due to its potent, broad-spectrum antimicrobial activity against Gram-positive, Gram-negative, and fungal pathogens. Silver acts by generating bacterial stress through direct contact and ions release: the antibacterial effect arises when the released Ag ions penetrate the membrane and condense DNA, leading to severe cell damage [83]. Smith et al. [88] pioneered a multicomponent tissue-engineering approach by integrating Ag nanoparticles into microporous 3D polycaprolactone (PCL) scaffold fabricated on titanium construct. These scaffolds were combined with hydrogel layers (HAMA or type I collagen (Col I)) and various antibacterial agents (Ag, TiO_2_ or chlorhexidine diacetate). A key finding of this work was the successful fabrication of a composite that achieved a tensile strength (29.62 MPa) comparable to the documented range for human skin (17-21 MPa) [89]. While the constructs demonstrated reduced bacterial viability *in vitro* (especially with chlorhexidine), this initial study focused on the complex composite material properties and did not isolate the specific antimicrobial contribution of silver, setting the stage for subsequent Ag-centered studies.

Subsequent investigations focused on defining the optimal therapeutic concentration for silver. Chimutengwende-Gordon et al. [90] electrochemically deposited different silver concentrations on hydroxyapatite coatings of titanium discs. They found that higher Ag content (100 mg/L of AgN $O_{3}$) effectively suppressed bacterial colonization of *S. aureus*, while lower silver concentrations maintained comparable antibacterial effects against *S. aureus* with reduced potential cytotoxicity for fibroblasts. This work was fundamental in identifying the optimal silver concentration that balances antimicrobial efficacy with soft tissue compatibility. More recently, Mishra et al. [91] developed a multifunctional composite membrane by embedding Ag nanoclusters (AgNCs∼3 nm) into a chitosan-polyethylen glycol (CS-PEG) matrix. This design not only hindered biofilm formation against both Gram-negative (*Escherichia coli*) and Gram-positive (*S. aureus*) bacteria, but also enhanced mechanical stability and enabled a sustainable release of the anti-inflammatory drug naproxen *in vitro*. Their results suggest a promising route toward multifunctional antimicrobial systems that integrate sustained antibacterial action with active, local theraupetic delivery.

Overall, while the development of such multifunctional systems has significantly widened the therapeutic window of antimicrobial metals, the long-term depletion of ion reservoirs and the risk of cumulative cytotoxicity remain critical bottlenecks.

## 5 Cell-based approach

Beyond material-based strategies, cell-based therapies represent a promising approach that has been investigated for enhancing tissue healing and implant integration. These strategies harness the regenerative potential of specific cell types, such as fibroblasts to facilitate matrix formation and bone marrow-derived mesenchymal stem cells (BMMSCs) to modulate the inflammatory response, with the goal of establishing a durable soft tissue seal at the skin-implant interface.

The foundational cell-based concept was pioneered by Kantrowitz et al. [92], in the context of a cardiac assist device. They hypothesized that fibroblasts pre-seeded on a porous implant surface could generate a dense collagen matrix capable of preventing epithelial downgrowth in percutaneous devices. This strategy, which aligns directly with the definition of cell therapy [93], used a porous fabric-covered device that successfully promoted *in situ* recruitment of native fibroblasts to form a seal, as observed in clinical trial [94]. While this pioneering work provided a robust proof-of-concept for soft tissue integration around a percutaneous device, only a limited number of subsequent studies have fully explored this alternative strategy for limb prostheses.

A pilot animal study conducted by Shevtsov et al. [95] evaluated the synergistic effect of surface topography and cell therapy by seeding dermal fibroblasts onto titanium pylons modified with titania nanotubes (TiO_2_-NT). In a rat model, this approach aimed to create a biological seal through the pre-establishment of a cellular interface. The results demonstrated that the combination of the 80 nm nanotubular diameter, which provided an optimal substrate for cell anchorage, and fibroblast pre-seeding accelerated the formation of a dense connective tissue layer. Over a 6-month follow-up, histological analysis revealed robust soft tissue ingrowth into the porous regions of the pylon, with no clinical signs of infection or marsupialization. This study confirmed that pre-seeding autologous cells onto nanostructured surfaces not only enhanced initial tissue attachment, but also provided a superior biological barrier against bacterial infiltration compared to smooth, non-seeded controls. Expanding upon these preliminary findings, Bolle et al. [96] investigated highly porous polycaprolactone scaffolds, fabricated by melt electro writing (MEW), which were pre-seeded with human dermal fibroblasts. By utilizing a 3D reconstructed human skin equivalent model, the authors demonstrated that pre-seeded scaffolds supported superior epidermal integration and significantly reduced epithelial downgrowth compared to unseeded controls. This improvement was attributed to the establishment of a robust dermal-like matrix within the MEW fibers prior to epidermalization, which provided the necessary mechanical and biochemical cues for keratinocyte anchorage. However, the study also highlighted that marsupialization was not prevented in all the cases, especially when the initial dermal maturation was insufficient. This suggests that, while scaffold architecture and cell seeding are critical, the pre-cultivation period and donor-specific fibroblast activity remain key variables for achieving a consistent biological seal.

In addition to fibroblast-based therapies, bone marrow mesenchymal stem cells (BMMSCs) have attracted considerable attention due to their regenerative capacity. Isackson et al. [97], evaluated BMMSCs pre-seeded on porous titanium implants in a rat model. Compared to untreated implants, the BMMSCs seeded constructs showed increased collagen deposition and accelerated transition from acute to chronic wound healing response. Although no significant differences were observed between treated and untreated groups in terms of neovascularization and overall tissue integration, the pre-seeded implants exhibited a reduced inflammatory response, evidenced by a thinner fibrous capsule and fewer macrophages and giant cells.

Collectively, these studies supported the potential of cell-based therapy to accelerate early healing and modulate the inflammatory responses, potentially improving the integration of porous transcutaneous implants. However, challenges remain regarding the consistency of outcomes and the scalability of these strategies for clinical application. Future research should focus on optimizing cell sourcing, delivery methods, and scaffold design to achieve reliable and durable soft tissue integration. Notably, the adoption of a 3D skin equivalent model [96] marks a conceptual shift from conventional monolayer (2D) culture toward more faithful testing platforms for skin architectures. This progression logically points to skin organoids [98] as a yet unexplored but promising *in vitro* framework for both pre-conditioning implant scaffolds and predicting *in vivo* integration outcomes. Ultimately, establishing a robust and durable dermal seal requires the synergistic integration of these cell-based approaches with optimized surface topographies and targeted antimicrobial strategies, bridging the gaps highlighted across all therapeutic modalities discussed.

## 6 Conclusions

The reviewed literature underscores the central challenge of achieving a stable, infection-resistant epidermal seal at the skin–implant interface, a critical requirement for the long-term success of percutaneous limb prostheses, particularly ITAP systems. Despite notable advances in surface engineering, including nano-topographical modification and biofunctional coatings, consistent long-term integration of the skin–implant interface remains a problem to solve. Clinical experience further indicates that strategies proven successful in dental implantology, such as the incorporation of basement-membrane components, like laminin-332, do not fully translate to the unique mechanical and biological demands of the lower limb's soft tissue environment.

While antimicrobial approaches, such as silver-based coatings, controlled-release systems, and antimicrobial peptides, have reduced early bacterial colonization and biofilm formation, they rarely provide durable protection. The paradigm has therefore shifted from passive defense to active immunomodulation. Recent advances demonstrate that nanoscale surface topography acts as a potent physical cue, steering macrophage differentiation away from the pro-inflammatory (M1) phenotype toward the pro-regenerative (M2) phenotype, thereby mitigating chronic foreign body reaction and epithelial downgrowth.

Similarly, although cell-based therapies, such as the application of mesenchymal stem cells (MSCs) [[95], [96], [97]], showed great promise in accelerating tissue healing and modulating inflammation, existing materials and surface treatments have yet to reconcile the complex interplay between epithelial downgrowth, dermal tissue attachment and host immune modulation consistently and at a clinically scalable level. These limitations collectively explain why infection and marsupialization remain major barriers to widespread clinical adoption. Despite most of the evidence discussed in this review remains preclinical, some clinical data are emerging, clarifying the translational potential of the above-mentioned strategies. The ITAP clinical trial addressed the issue related to the surface-engineered percutaneous interface in humans, but focusing on bone-implant interface. However, the results highlighted how a hydroxyapatite coating alone is not sufficient to guarantee long-term interface stability, with progressive degradation leading to infection and implant failure [99], supplying early outcomes on bioactive coating effect. Regarding antimicrobial coatings, prospective clinical studies on silver-coated megaprostheses reported consistent reductions in periprosthetic infection rates when compared to uncoated titanium implants — from 17.6% to 5.9% [100] and from 16.7% to 8.9% [101] — providing a human-level confirmation that antimicrobial metallic coatings can be effective. More recently, antibiotic-loaded hydrogel coatings have shown promising results in transcutaneous osseointegrated prostheses [102]. Overall, a clinical trial on surface-engineered skin–implant interfaces for limb prostheses is still lacking, but this translational basis could become helpful in establishing new protocols in this direction. The critical gap is therefore not only the limited availability of clinical evidence, but also the lack of standardized, comparative, and clinically relevant models to assess the performance of individual and combined strategies.

### 6.1 Future perspectives and challenges

Future research should move beyond the isolated evaluation of individual strategies toward integrated models that explicitly address the competing requirements of the skin–implant interface. Combining antimicrobial defense with direct biological stimulation, through surfaces that promote ECM formation, keratinocyte anchorage, and fibroblast proliferation, offers a promising direction. Hybrid bioactive systems capable of simultaneously supporting antimicrobial defense and robust M2-driven immune homeostasis, as recently demonstrated by NIR-responsive nanoscale architectures [56], highlight the potential of multifuncional designs.

The next challenge is therefore to determine whether highly porous bioactive interfaces can be engineered to provide synergistic, rather than merely additive, effects by simultaneously promoting soft-tissue sealing, controlling bacterial colonization, and guiding a pro-regenerative immune response.

## Author contribution

Ilaria Sergio: acquisition of data, interpretation of data, Drafting the manuscript, Approval of the version of the manuscript to be published (the names of all authors must be listed).

Rosalaura Caforio: acquisition of data, interpretation of data, Drafting the manuscript, Approval of the version of the manuscript to be published (the names of all authors must be listed).

Laura Sercia: acquisition of data, interpretation of data, Drafting the manuscript, Approval of the version of the manuscript to be published (the names of all authors must be listed); Giuseppe Gigli: Approval of the version of the manuscript to be published (the names of all authors must be listed); Francesca Gervaso: Conception and design of study, revising the manuscript critically for important intellectual content, Approval of the version of the manuscript to be published (the names of all authors must be listed); Alessandro Polini: revising the manuscript critically for important intellectual content, Approval of the version of the manuscript to be published (the names of all authors must be listed); Francesca Scalera: Conception and design of study, acquisition of data, interpretation of data, Drafting the manuscript, revising the manuscript critically for important intellectual content, Approval of the version of the manuscript to be published (the names of all authors must be listed).

## Declaration of generative AI in scientific writing

No generative artificial intelligence (AI) or AI-assisted technologies were used in the preparation of this manuscript.

## Funding statement

Authors would also like to thank the Italian Ministry of Research (MUR) for supporting this work through the complementary actions to the NRRP “Fit4MedRob” Grant (PNC0000007, CUP B53C22006960001) and 10.13039/501100009886Regione Puglia through the “Tecnopolo per la medicina di precisione” (TecnoMed Puglia)-Regione Puglia: 10.13039/501100019945DGR n.2117 del 21/11/2018, CUP: B84I18000540002.

## Declaration of competing interest

The authors declare that they have no known competing financial interests or personal relationships that could have appeared to influence the work reported in the manuscript entitled “Enhancing Skin-Implant Integration in Lower-Limb Transcutaneous Prostheses: From Interface Biology to Bioactive, Antimicrobial and Cell-Based Strategies.”
